# SPINK7 Recognizes Fungi and Initiates Hemocyte-Mediated Immune Defense Against Fungal Infections

**DOI:** 10.3389/fimmu.2021.735497

**Published:** 2021-09-17

**Authors:** Zhaoming Dong, Lingna An, Mengyao Lu, Muya Tang, Haiqin Chen, Xuan Huang, Yong Hou, Guanwang Shen, Xiaolu Zhang, Yan Zhang, Qingyou Xia, Ping Zhao

**Affiliations:** ^1^State Key Laboratory of Silkworm Genome Biology, Southwest University, Chongqing, China; ^2^Biological Science Research Center, Southwest University, Chongqing, China; ^3^Chongqing Key laboratory of Sericultural Science, Chongqing Engineering and Technology Research Center for Novel Silk Materials, Southwest University, Chongqing, China

**Keywords:** protease inhibitor, SPINK, cell immunity, hemocyte, nodulation, encapsulation, insect, kazal

## Abstract

Serine protease inhibitors of Kazal-type (SPINKs) were widely identified in vertebrates and invertebrates, and played regulatory roles in digestion, coagulation, and fibrinolysis. In this study, we reported the important role of SPINK7 in regulating immune defense of silkworm, *Bombyx mori*. SPINK7 contains three Kazal domains and has 6 conserved cysteine residues in each domain. Quantitative real-time PCR analyses revealed that *SPINK7* was exclusively expressed in hemocytes and was upregulated after infection with two fungi, *Saccharomyces cerevisiae* and *Candida albicans*. Enzyme activity inhibition test showed that SPINK7 significantly inhibited the activity of proteinase K from *C. albicans*. Additionally, SPINK7 inhibited the growth of three fungal spores, including *S. cerevisiae*, *C. albicans*, and *Beauveria bassiana*. The pathogen-associated molecular patterns (PAMP) binding assays suggested that SPINK7 could bind to β-D-glucan and agglutinate *B. bassiana* and *C. albicans*. *In vitro* assays were performed using SPINK7-coated agarose beads, and indicated that SPINK7 promoted encapsulation and melanization of agarose beads by *B. mori* hemocytes. Furthermore, co-localization studies using immunofluorescence revealed that SPINK7 induced hemocytes to aggregate and entrap the fungi spores of *B. bassiana* and *C. albicans*. Our study revealed that SPINK7 could recognize fungal PAMP and induce the aggregation, melanization, and encapsulation of hemocytes, and provided valuable clues for understanding the innate immunity and cellular immunity in insects.

## Introduction

The insect innate immune system mainly functions *via* immune recognition, followed by signaling cascade activation and induction of immune effectors. Activation of innate immunity by microorganisms is initiated by pathogen-associated molecular patterns (PAMPs), including peptidoglycan (PGN), lipopolysaccharide (LPS), and β-1,3-glucan, which are bacterial and fungal cell wall and membrane components. In the silkworm *Bombyx mori*, PAMPs are recognized by pattern-recognition receptors (PRRs) (1), which are mainly peptidoglycan recognition proteins (PGRPs), β-1,3-glucan recognition proteins (βGRPs), and lectins. During humoral immunity, PGRPs and βGRPs induce signal amplification through proteolytic cascade reactions, resulting in the production of microbicidal substances such as antimicrobial peptides (AMPs), lysozyme, and melanin (2). For cellular immunity, lectins, hemocytins, and thioester-containing proteins induce phagocytosis, nodulation, and encapsulation, which are mediated by hemocytes (1, 3).

In 2014, Zhang et al. identified 85 immunity-related proteins in the silkworm hemolymph, twenty-seven of which were protease inhibitors (4). Furthermore, Chen et al. found that 102 proteins were significantly upregulated in silkworm hemolymph after bacterial infections, twelve of which were protease inhibitors (5). In 2012, Zhao et al. observed that 8, 4, 3, and 3 protease inhibitor genes were upregulated after infection with *Escherichia coli*, *Bacillus bombysepticus*, *Beauveria bassiana*, and *Bombyx mori* nuclear polyhedrosis virus, respectively (6). These observations indicate that protease inhibitors may play important roles in insect innate immunity. Currently, more than 10 protease inhibitor families have been identified in the silkworm (6), including serpin, Kunitz_BPTI, Kazal, TIL, amfpi, Bowman-Birk, Antistasin, WAP, Pacifastin, and alpha-macroglobulin. Protease inhibitors from 9 families contain numerous cysteine residues (6). For example, serine protease inhibitor of Kazal-type (SPINK) usually contains six cysteines to form three intramolecular disulfide bonds (6).

SPINKs were first discovered in the human pancreas and described by Louis A. Kazal (7), and then were widely identified in animals, including mammals (8, 9), birds (10–12), insects (13), and crayfish (14), which played a variety of regulatory roles in digestion, coagulation (15), and fibrinolysis (9, 16). Generally, SPINK contains one or more Kazal domains, each of which has approximate molecular size of 5-7 kDa (16, 17). Some studies provide evidence that SPINKs might be involved in immune defense. For example, a human skin-derived SPINK9 exhibited killing activity against *Escherichia coli* (18). A carp seminal plasma SPINK2 showed bacteriostatic activity against *E. coli*, *Lactobacillus subtilis* and *Aeromonas hydrophila* (19). A crayfish hemocyte SPINK (hcPcSPI2) showed bacteriostatic activities on the growth of *Bacillus subtilis* and *Bacillus thuringiensis* (14). A jellyfish-derived SPINK from *Cyanea capillata* inhibited the growth of various gram-positive bacteria and gram-negative bacteria (20). A bee venom SPINK (AcKTSPI) from *Apis cerana* showed antibacterial activity against gram-positive bacteria and antifungal activity against pathogenic fungi (21).

Ten SPINKs have been identified in the silkworm, and were designated as BmSPI59-68 (SPINK1-10) (6). BmSPI65 (SPINK7) and BmSPI66 (SPINK8) showed significant upregulation in the hemolymph after infection with Gram-negative *Escherichia coli* and Gram-positive *Staphylococcus aureus* (5). In addition, a new silkworm SPINK was identified, which could be named as SPINK11, and was reported as a specific subtilisin inhibitor and hypothesized as a bacteriostatic protein (22). In the present study, we focused on the silkworm SPINK7, and found it could recognize invasive fungi and promote the aggregation of hemocytes to encapsulate fungi. This study provided new insights regarding innate immunity and cellular immunity in the insects.

## Materials and Methods

### Sample Preparation

*B. mori* strain Dazao silkworms were reared on fresh mulberry leaves at room temperature and a 12 h light/dark cycle. Three fungal species—*C. albicans*, *B. bassiana*, and *S. cerevisiae* (BeNa Culture Collection, China)—were cultured on potato dextrose agar medium for 15 days at 30°C. Fungal conidia were washed in distilled water containing 0.05% Tween-80 (v v^-1^), filtered with sterile absorbent cotton, washed three times with Milli Q water, and diluted to 1 × 10^5^ conidia mL^-1^ with 20 mM phosphate-buffered saline (PBS) buffer (pH 7.5).

Fungal spore liquid (10 µL) was injected into each larva on day 3 of the fifth instar. Larvae injected with sterile PBS were used as the control group. The hemolymph of 10 silkworms was collected at 6, 12, 18, and 24 h after injection. Then, the hemolymph was centrifuged for 10 min at 1,000*g* at 4°C to separate hemocytes from the serum. Seven larval tissues were collected on day 7 of the fifth instar, including head, integument, fat body, hemocyte, gonad, midgut, and silk gland. All samples were stored at -80°C until use.

### Sequence Analysis

The SPINK7 sequence was downloaded from the NCBI database. Forecast analysis of protein amino acid composition, molecular weight and isoelectric point information were using online software Protparam (http://web.expasy.org/protparam/) and ProtScale (http://web.expasy.org/protscale/). Prediction of signal peptide was carried out using SignalP 4.1 Server (http://www.cbs.dtu.dk/services/SignalP-4.1/). Multiple alignments of the Kazal domains were conducted using the program CLUSTAL-W. The active site of individual domain was determined through the alignments of the homolog sequence.

### Protein Expression and Purification

The *SPINK7* gene coding region (without signal peptides) was PCR-amplified using a sense primer (5′- CGC GGA TCC TAT CCA CCA AGC TGT GCG TG -3′) and an antisense primer (5′- ATT TGC GGC CGC CAG GGG TCG TAT TCC GTG GT -3′). The purified PCR product was inserted into the pET28a vector and transformed into *E. coli* strain BL21 (TransGen Biotech, China). The *E. coli* cells were induced with 0.1 mM IPTG and then lysed in binding buffer (20 mM Tris-HCl, 200 mM NaCl, pH 7.5) by sonication. After centrifugation at 12,000*g* for 25 min, the supernatants were loaded onto a nickel affinity chromatography column, which was equilibrated with binding buffer. The bound protein was eluted with gradient elution buffer (0-500 mM imidazole in binding buffer) and then loaded onto a HiLoad 16/60 Superdex 200 column (GE Healthcare, USA) pre-equilibrated with 20 mM PBS (pH 7.5). The purified SPINK7 protein was separated by 12% SDS-PAGE and the protein sample was stored at -80°C.

### Assay for Inhibitory Activity Against Protease

Assay for inhibitory activity against protease was performed as previously described with modification (23–27). Trypsin, α-chymotyrpsin, pronase, subtilisin, thrombin were purchased from Sigma-Aldrich (St. Louis, USA), and proteinase K and papain were purchased from Sangon Biotech (Shanghai, China). SPINK7 (100 μg) was preincubated with 10 μg protease in 100 μL Fluoro assay buffer (100 mM Tris-HCl and 20 mM CaCl_2_, pH 7.5) for 30 min at 37°C. Then, 100 μL FITC-casein substrate buffer (G-Biosciences, USA) was added and incubated at 37°C in the dark for 1 h. The fluorescence was measured at 485/535 nm (excitation/emission). Protease inhibition by SPINK7 was assessed using the following formula: % residual activity = (residual enzyme activity/enzyme activity without inhibitor) × 100. Three independent replicates were performed for each experiment. Student’s t test was used to evaluate statistical significance. These data were analyzed by GraphPad Prism 5 software.

### Quantitative Real-Time PCR

The qPCR was performed with qTOWER 2.2 PCR Thermal Cycler (Analytikjena, Germany) using SYBR Premix Ex Taq II (TaKaRa, Japan) and SPINK7-specific primers (forward primer: 5’-cct ttg cgc aga cga agt -3’, reverse primer: 5’-cac gtg cac gaa ggg tat t-3’). The qPCR program was performed as follows: initial denaturation at 95°C for 30s, followed by 40 cycles of denaturation at 95°C for 20 s, annealing at 60°C for 60 s, and extension at 72°C for 35 s. Relative expression was calculated from *SPINK7* and *sw22934* (Translation initiation factor 4A, Uniprot ID: Q285R3) (28) gene expression values (recorded as Ct and Csw, respectively). The relative *SPINK7* expression (Cr) was then calculated using the following formula: Cr=2^-△(Ct−Csw)^. These data were analyzed by GraphPad Prism 5 software.

### Western Blot Analysis

Serum samples were separated using 12% SDS-PAGE followed by electrotransfer to PVDF membrane (GE Healthcare, USA). The membrane was blocked with 5% BSA in TBST buffer (50 mM Tris-HCl, 500 mM NaCl, and 0.2% Tween 20, pH 8.0) for 2 h, and then incubated with rabbit anti-SPINK7 or anti-storage protein 2 (SP2) for 1.5 h at 25°C. After washing three times with TBST, the membranes were incubated with horseradish peroxidase-conjugated goat anti-rabbit IgG (Beyotime, China) at 25°C for 1h and detected using ECL reagent (GE Healthcare, USA).

### Assay for Inhibitory Activity Against Fungal Growth

Growth inhibition assays were carried out in 96-well plates (Corning, USA). Each well was added with 200 μL potato liquid medium containing fungal conidia at a final concentration of 1 × 10^5^ conidia mL^-1^. The culture medium contained chloramphenicol (25 μg mL^-1^) to prevent bacterial infection. The protein concentrations of SPINK7 used in this section were 0.1 mg mL^-1^ and 0.5 mg mL^-1^, respectively. PBS and BSA were used as negative controls, while EDTA was used as a positive control. Microplates were incubated at 30°C with shaking at 45 rpm. Fungal growth was observed by monitoring the absorbance at 595 nm after culturing for 0, 12, 24, 36, and 48 h. Student’s t test was used to evaluate statistical significance. Three independent replicates were performed for each experiment. These data were analyzed by GraphPad Prism 5 software.

### Experiment of Fungal Cell Death Detection

Amphotericin B can bind to sterols in the fungal cell membrane and rupture the cell membrane. Further, propidium iodide (PI) can bind to the DNA of dead cells and emit red fluorescence, but cannot pass through healthy cell membranes. Thus, PI fluorescence can indicate whether SPINK7 causes fungal death. 50 μL SPINK7 solution (5 μg μL^-1^) was preincubated with 450 μL *B. bassiana* or *C. albicans* spore solution in a final concentration of 1 × 10^5^ conidia mL^-1^ for 2 h at room temperature. Then, 12.5 μL amphotericin B (100 μg μL^-1^) was added to 487.5 μL spore solution in a final concentration of 1 × 10^5^ conidia mL^-1^ and incubated for 2 h at room temperature. 50 μL PBS was used instead of SPINK7 solution as the control group. Then, fungal cells were stained with 1:1000 PI fluorescence (1 mg/mL) in the dark for 10 min. Finally, 10 μL solution was mounted on a microscope slide and coverslipped. The slides were observed under a confocal microscope (LSM 880; Zeiss, Germany). Three independent replicates were performed for each experiment.

### PAMPs Binding Assay

Briefly, 40 μg β-D-glucan (Sigma–Aldrich, USA) and mannan (Sigma–Aldrich, USA) were dissolved in coating solution (0.5 M carbonate buffer, pH 9.8), added to a 96-well microtiter plate, and incubated at 4°C overnight. The uncoated PAMPs in the 96-well plate was washed three times with PBS (10 mM Na_2_HPO_4_, 2 mM KH_2_PO_4_, 137 mM NaCl, 2.7 mM KCl, pH 7.4) and incubated with blocking buffer (3% BSA and 10% normal goat serum in PBS) for 30 min at 37°C. After washing the 96-well plate with PBS, 0 mg mL^-1^, 0.1 mg mL^-1^, 0.3 mg mL^-1^, and 0.5 mg mL^-1^ SPINK7 in PBS were added and incubated at 25°C for 3 h. After washing the 96-well plate 3 times (10 min each) with PBST (PBS containing 0.1% Tween), 1:1000 rabbit anti-SPINK7 (AbMax Biotech, China) in PBS containing 1% BSA was added and incubated at 37°C for 1 h. The wells were washed three times for 10 min each with PBST. Next, 1:1000 anti-rabbit IgG (H+L) (Beyotime, China) was added and incubated at 37°C for 1 h. Then, 100 µL TMB (3,3’,5,5’-Tetramethylbenzidine) was added to quench the reaction. Absorbance was measured at OD_450_ using a microplate reader. Non-immune rabbit serum was used as a negative control, and empty wells were used as blanks. Each experiment was repeated in triplicate. Samples with (P_sample_ - B_blank)/_(N_negative_ - B_blank_) > 2.1 were considered positive (29). Student’s t-test was used to evaluate statistical significance. Three independent replicates were performed for each experiment. These data were analyzed by GraphPad Prism 5 software.

### Competitive PAMPs and Fungal Spores Binding With SPINK7

20 μL SPINK7 solution (5 μg μL^-1^) was preincubated with 200 μL β-D-glucan solution (80 μg mL^-1^ in PBS) or mannan solution (80 μg mL^-1^ in PBS) for 1 h at room temperature. Then, each sample was incubated with 200 μL *C. albicans* spore solution in a final concentration of 1 × 10^5^ conidia mL^-1^ for 1 h at room temperature. 20 μL PBS was used instead of SPINK7 solution as the control group. The spores were incubated with blocking buffer (1% BSA in PBS) at 25°C for 1 h, and then incubated with 1:1000 rabbit anti-SPINK7 (AbMax Biotech, China) in the blocking buffer for 1.5 h at 25°C. Then, samples were washed three times in PBS, and incubated with 1:1000 Cy3-labeled goat anti-rabbit IgG (Beyotime, China) in blocking buffer. Finally, after washing twice with PBS, the spores were observed with a fluorescence microscope (Olympus BX51, Japan). Three independent replicates were performed for each experiment.

### *In Vitro* Encapsulation and Melanization

*In vitro* encapsulation and melanization assays were performed as previously described (29, 30), with slight modifications. Ni-NTA agarose beads (Qiagen, Germany) were equilibrated in PBS containing 5 mM CaCl_2_. Recombinant SPINK7 or tubulin (as a control protein) was incubated overnight with agarose beads in a 1.5 mL tube at 4°C with shaking. Additional protein was added until agarose bead binding was saturated (excess proteins were detected in the remaining supernatant). Generally, 200 μL agarose beads can bind up to 1 mg recombinant SPINK7. Protein-coated beads were washed four times for 5 min with PBS and suspended in PBS at 80-100 beads per microliter (about 1 μg protein per microliter beads).

A 48-well cell culture plate (Falcon) was treated with 1% agarose (ISC Bioexpress). Hemolymph collected from 10 silkworm larvae was combined with 200 μL Grace’s Insect Medium (Gibco, Waltham, MA, USA) supplemented with 10% (v v^-1^) FBS (Bio Basic Inc., Tornoto, Canada). The diluted hemolymph was added to each well of the agarose-coated plate. Hemocytes were allowed to settle for at least 10 min. Then, 1 μL (80-100 beads) protein-coated agarose beads was added to each well, and the plate was incubated at 25°C. Encapsulation and melanization of the agarose beads were observed after 6 and 24 h incubation, respectively. For the recombinant protein, assay was performed in three different wells.

To test whether *in vitro* encapsulation and melanization can be blocked by rabbit polyclonal antiserum specific for SPINK7, 5 μL protein-coated beads (about 5 μg of recombinant proteins on the surface) were placed in a microcentrifuge tube containing 50 μL 1:500 rabbit anti-SPINK7. Then, the samples were incubated overnight at 4°C with shaking. The beads were washed with PBS 3 times and resuspended in 5 μL PBS. The plate was observed with a fluorescence microscope (Olympus BX51, Japan). Three independent replicates were performed for each experiment.

### Immunofluorescence Assay

Overlay experiments were performed on hemocyte monolayers prepared by seeding 5×10^3^ hemocytes collected in phenylthiourea onto poly-L-lysine-treated glass slides. About 10 μL protein sample (5 mg mL^-1^) and 10 μL heat-killed spores (1×10^5^ conidia mL^-1^) labeled with Calcofluor White M2R (MKbio, China) were added to the hemocyte monolayer and incubated at 25°C for 2 h (31). We used 5 μg μL^-1^ BSA and 5 μg μL^-1^ hdSPINK7 (heat denatured SPINK7) to replace SPINK7 as a control experiment. Then, the hemocytes were washed once with PBS and fixed with 4% paraformaldehyde at 25°C for 5 min. After washing twice with PBS, cells were permeabilized with 0.1% Triton X-100 in PBS. After blocking with 1% BSA and 10% normal goat serum in PBS at 25°C for 1 h, 1:500 mouse anti-tubulin antibody (Beyotime, China) and 1:500 anti-SPINK7 rabbit antibody (AbMax Biotech, China) in PBS containing 1% BSA were incubated with the cells for 1.5 h at 25°C. The cells were washed three times in PBS and incubated with 1:500 Cy3-Labeled goat anti-rabbit IgG and 1:500 Alexa Fluor 488-labeled goat anti-Mouse IgG (H+L) (Beyotime, China) in PBS containing 1% BSA for 1 h at 25°C. Finally, after washing twice with PBS, the slides were observed with a fluorescence microscope (Olympus BX51, Japan). Three independent replicates were performed for each experiment.

## Results

### Bioinformatics Analysis of SPINK7

**Figure 1:**
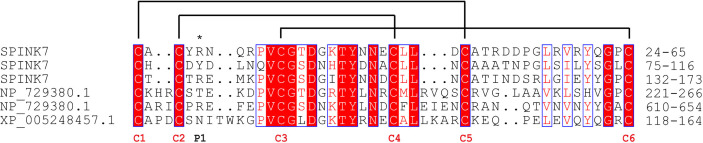
Sequence alignment of SPINKs from *Bombyx mori*, *Drosophila melanogaster* and *Homo sapiens*. *B. mori* SPINK7, GenBank accession No. XP_004924430.1; *D. melanogaster* SPINK, GenBank accession No. NP_729380.1; *H. sapiens* SPINK, GenBank accession No. XP_005248457.1. The conserved cysteine residues and reactive sites are indicated with red “C” and an asterisk, respectively. Disulfide linkages are indicated with solid lines.

The open reading frame of the *SPINK7* gene encodes a 194-amino acid protein. The predicted signal peptide of SPINK7 is the 19 amino acid N-terminal sequence, indicating that SPINK7 might be extracellularly secreted. The mature protein has a theoretical molecular weight (MW) of 18.8 kDa and a isoelectric point (pI) of 4.7. SPINK7 was predicted to have three Kazal domains, each of which contains 6 conserved cysteine residues, which are responsible for intramolecular disulfide bridge formation (27, 32). Multiple sequence alignment showed that the Kazal domain of silkworm SPINK7 has high homology (approximate 40%) with those of fruit fly and human proteins (**Figure 1**). Relative high sequence conservation was observed around the third and fourth cysteine residues in the Kazal domain. P1 residues in the three Kazal domains of SPINK7 were predicted as Arg^28^, Tyr^79^, and Arg^136^, respectively (**Figure 1**), which locates in amino-termini of the cleavage site and determines the specific inhibitory activity against proteases. Since Arg and Tyr are cleavage sites for trypsin and chymotrypsin, respectively, we speculate that SPINK7 may have inhibitory activity against trypsin and chymotrypsin.

### Prokaryotic SPINK7 Expression and Anti-Protease Activity Assay

**Figure 2:**
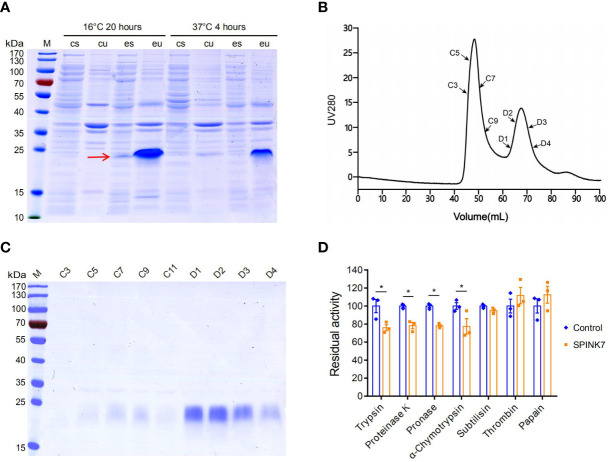
SPINK7 prokaryotic expression and activity. **(A)** SDS-PAGE analyses of recombinant SPINK7 protein. *E. coli* BL21 were induced with 0.1 mM IPTG at 16°C for 20 h or at 37°C for 4 h. Uninduced *E. coli* BL21 were used as the control. “M” indicates the molecular weight standards. “cu” and “eu” represent the insoluble component in the control and experimental samples, respectively. “cs” and “es” represent the supernatant proteins in the control and experiment samples, respectively. The arrow shows soluble SPINK7 protein. **(B)** Purification of the recombinant SPINK7 by gel filtration after affinity column chromatography. **(C)** SDS-PAGE analyses of SPINK7 after gel filtration. “C” and “D” represent the third and fourth elution peak, respectively. “C1-11” represents the first to eleventh tubes of protein solution collected from the third peak, whereas “D1-4” represents the first to fourth tubes of protein solution collected from the fourth peak. **(D)** Detection of recombinant SPINK7 inhibitory activities against various proteases. Bars on graph indicate standard error of the mean (n = 3). Student’s t-test: *P < 0.05.

To obtain SPINK7 protein for biochemical analysis, SPINK7 was expressed using a prokaryotic *E. coli* expression system. As shown in **Figure 2A**, a dominant protein band less than 25 kDa was detected in both of the soluble and insoluble fraction after incubation at 16°C for 20 h. This result is consistent with its theoretical molecular weight of 18.8 kDa. Molecular sieve and SDS-PAGE results showed that the D1-D4 peak contained high-purity His-tagged recombinant SPINK7 protein (**Figures 2B, C**). Western blot showed the purified protein was indeed SPINK7 (**Figure S1**). Analysis of SPINK7 inhibitory activity against seven proteases revealed that SPINK7 had significant inhibitory activity against trypsin and chymotrypsin from animals, pronase from *Streptomyces griseus*, and proteinase K from *Candida albicans.* However, inhibitory activity against subtilisin, thrombin, and papain was not detected (**Figure 2D**).

### SPINK7 Upregulation in Hemocytes From Fungal Infection

**Figure 3:**
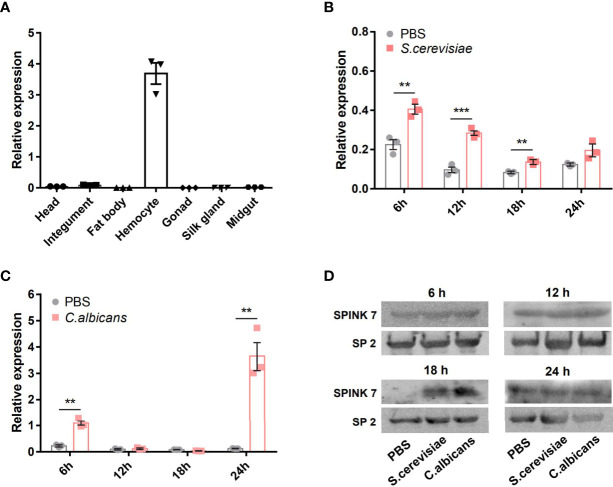
SPINK7 expression profiles. The expression patterns of SPINK7 in seven tissues **(A)**, including head, integument, fat body, hemocytes, gonad, silk gland, and midgut. SPINK7 mRNA expression levels in larval hemocytes at 6, 12, 18, and 24 h after infection with **(B)** *S. cerevisiae* and **(C)** *C. albicans*. Vertical bars represent the mean ± standard error of the mean (n = 3). Student’s t-test: ***P < 0.001 and **P < 0.01. SPINK7 protein in larval serum was detected by Western blot **(D)** at 6, 12, 18, and 24 h after infection with *S. cerevisiae* and *C. albicans*. The storage protein 2 was used as a loading control.

The expression profile of *SPINK7* in various tissues was investigated by qRT-PCR. Analysis of day 7 of fifth instar larvae showed that *SPINK7* was highly and exclusively expressed in hemocytes (**Figure 3A**). The qPCR was also used to investigate *SPINK7* expression after infection with two kinds of fungi: *S. cerevisiae* and *C. albicans*. *SPINK7* expression was significantly upregulated at 6, 12, and 18 h in hemocytes after *S. cerevisiae* infection (**Figure 3B**), and significantly upregulated at 6 and 24 h after *C. albicans* infection (**Figure 3C**). Western blot analysis was used to detect SPINK7 protein in the cell-free hemolymph after *S. cerevisiae* and *C. albicans* infection. SPINK7 protein was found to be upregulated at 18h after *S. cerevisiae* or *C. albicans* infection (**Figure 3D**). These results implied that SPINK7 may be secreted by hemocytes and involved in the immune defense against fungi.

### Antifungal Activity of SPINK7

**Figure 4:**
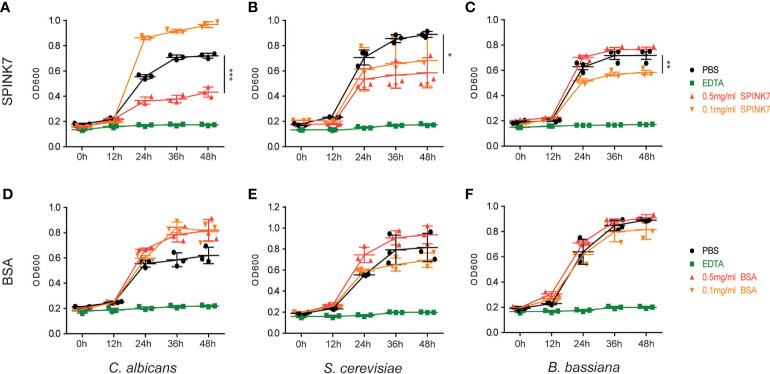
Antifungal activity of SPINK7. The inhibitory effect of SPINK7 on the growth of **(A)** *C. albicans*, **(B)** *S. cerevisiae*, and **(C)** *B. bassiana*. The inhibitory effect of BSA on the growth of **(D)** *C. albicans*, **(E)** *S. cerevisiae*, and **(F)** *B. bassiana*. The bar graph shows the SPINK7-mediated inhibitory effect on fungal growth at 48 h. Bars indicate standard error (n = 3). Student’s t-test: *P < 0.05, **P < 0.01, ***P < 0.001.

To evaluate SPINK7 antifungal activity, *S. cerevisiae*, *C. albicans*, and *B. bassiana* spores were incubated with 0.5 mg mL^-1^, and 0.1 mg mL^-1^ SPINK7. The fungal spore growth curve was monitored by UV spectrophotometry. SPINK7 inhibition of fungal growth at 48 h was shown in **Figure 4** by bar graph. The results showed that 0.5 mg mL^-1^ SPINK7 could significantly inhibit the growth of *C. albicans* (**Figure 4A**) and *S. cerevisiae* spores (**Figure 4B**). Further, 0.1 mg mL^-1^ protein could significantly inhibit *B. bassiana* spore growth (**Figure 4C**). We used BSA as a negative control, and confirmed that BSA had no significant inhibitory effect on the *C. albicans* (**Figure 4D**), *S. cerevisiae* (**Figure 4E**), and *B. bassiana* (**Figure 4F**) growth. These results clearly demonstrated the fungistatic effect of SPINK7.

### Interaction Between SPINK7 and *B. bassiana* Spores

**Figure 5:**
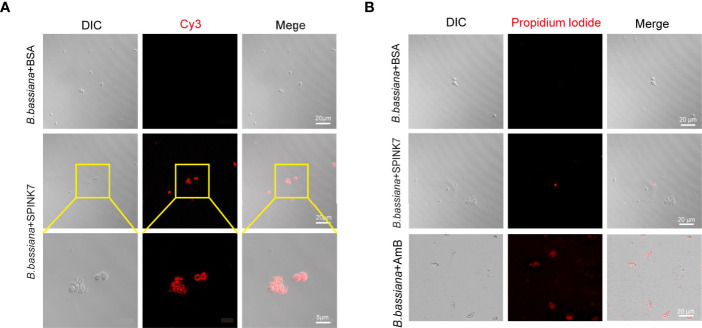
Interaction between SPINK7 and *B. bassiana* spores. **(A)** Immunofluorescence visualization of SPINK7 binding on the surface of *B. bassiana* spores. SPINK7 was preincubated with *B. bassiana* spores, and then incubated with anti-SPINK7 antibodies followed by a secondary antibody labeled with Cy3 (red). **(B)** Photomicrographs of *B. bassiana* spores stained with propidium iodide after treatment with amphotericin B (AmB) or BSA or SPINK7. Red (propidium iodide) indicates cells with ruptured membranes.

By incubating SPINK7 with *B. bassiana* spores, we found that SPINK7 binds *B. bassiana* spores to aggregate them together (**Figure 5A**). As control, BSA did not aggregate *B. bassiana* spores (**Figure 5A**). Furthermore, we observed that nearly all the *B. bassiana* spores were stained with propidium iodide (PI) after treatment by amphotericin B (33), which killed fungi by breaking spore membrane and allowed the fungal nucleic acid to leak out of the nucleus (**Figure 5B**). However, only a few of spores was stained with PI after SPINK7 treatment, and no spore was stained with PI after BSA treatment (**Figure 5B**), indicating that SPINK7 had weak destructive activity on cell membrane of *B. bassiana* and BSA did not break the spore membrane.

### Interaction Between SPINK7 and *C. albicans*

**Figure 6:**
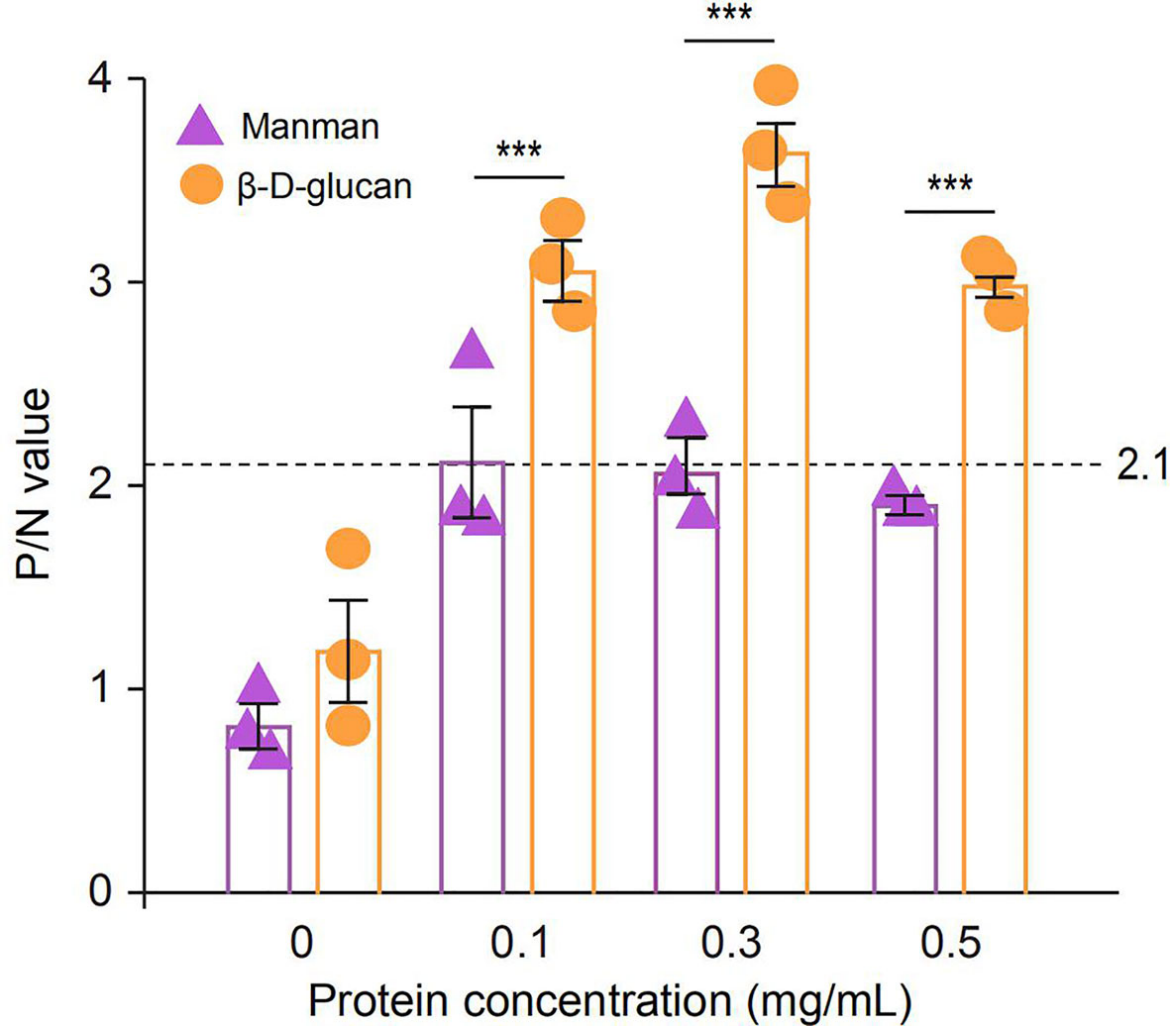
ELISA analysis of the interaction between recombinant SPINK7 and fungal PAMPs. Plates were coated with two fungal PAMPs (β-D-glucan and mannan), and then incubated with SPINK7 at 25°C for 3h. After stopping the TMB color reaction, the plates were read at OD_450_ using a microplate reader. The negative control is unimmunized serum, and empty wells were used as blanks. Samples with (P_sample_ - B_blank)/_(N_negative_ - B_blank_) > 2.1 were considered positive. Student’s t-test: ***P < 0.001. Bars indicate standard error of the mean (n = 3).

To identify the PAMP binding spectrum of SPINK7, binding assays were performed using two fungal polysaccharides, β-D-glucan and mannan. Binding activity was recorded as P/N at OD_450_, and samples with P/N > 2.1 indicated positive binding. The results showed that SPINK7 exhibited positive binding with β-D-glucan at concentration of 0.1-0.5 mg/mL but negative binding with mannan at concentration of 0-0.5 mg/mL (**Figure 6**).

**Figure 7:**
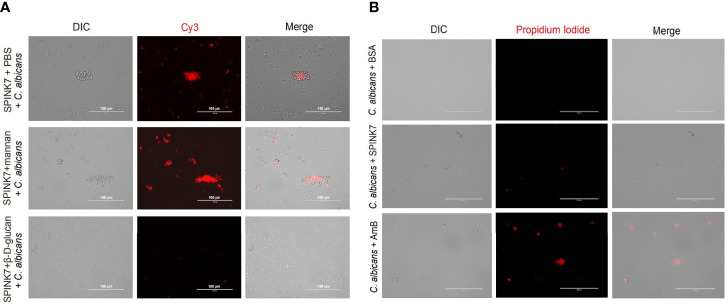
Interaction between SPINK7 and *C. albicans*. **(A)** Immunofluorescence visualization of SPINK7 binding to *C. albicans*. SPINK7 was preincubated with PBS, mannan, and β-D-glucan, respectively, and then incubated with *C. albicans* spores. Slides were then incubated with anti-SPINK7 antibodies followed by a secondary antibody labeled with Cy3 (red). **(B)** Photomicrographs of *C. albicans* stained with propidium iodide after treatment with amphotericin B (AmB) or BSA or SPINK7. Red (propidium iodide) indicates cells with ruptured membranes.

SPINK7 was preincubated with excess β-D-glucan and mannan, and then incubated with *C. albicans* (**Figure 7A**). The results showed that SPINK7 could bind to *C. albicans* after incubation with mannan (**Figure 7A**), because SPINK7 could not bind to mannan. SPINK7 lost binding ability with *C. albicans* after incubation with excess β-D-glucan, because β-D-glucan completely occupied the polysaccharide-binding sites of SPINK7. These results suggest that SPINK7 could bind to the fungi to play antifungal roles.

Furthermore, we observed that *C. albicans* were stained with PI after treatment by amphotericin B (31), and only a few of spores was stained with PI after SPINK7 treatment, and no spore was stained with PI after BSA treatment (**Figure 7B**), indicating that SPINK7 had weak destructive activity on the cell membrane of *C. albicans*.

### SPINK7 Promotes Hemocyte Aggregation and Melanization

**Figure 8:**
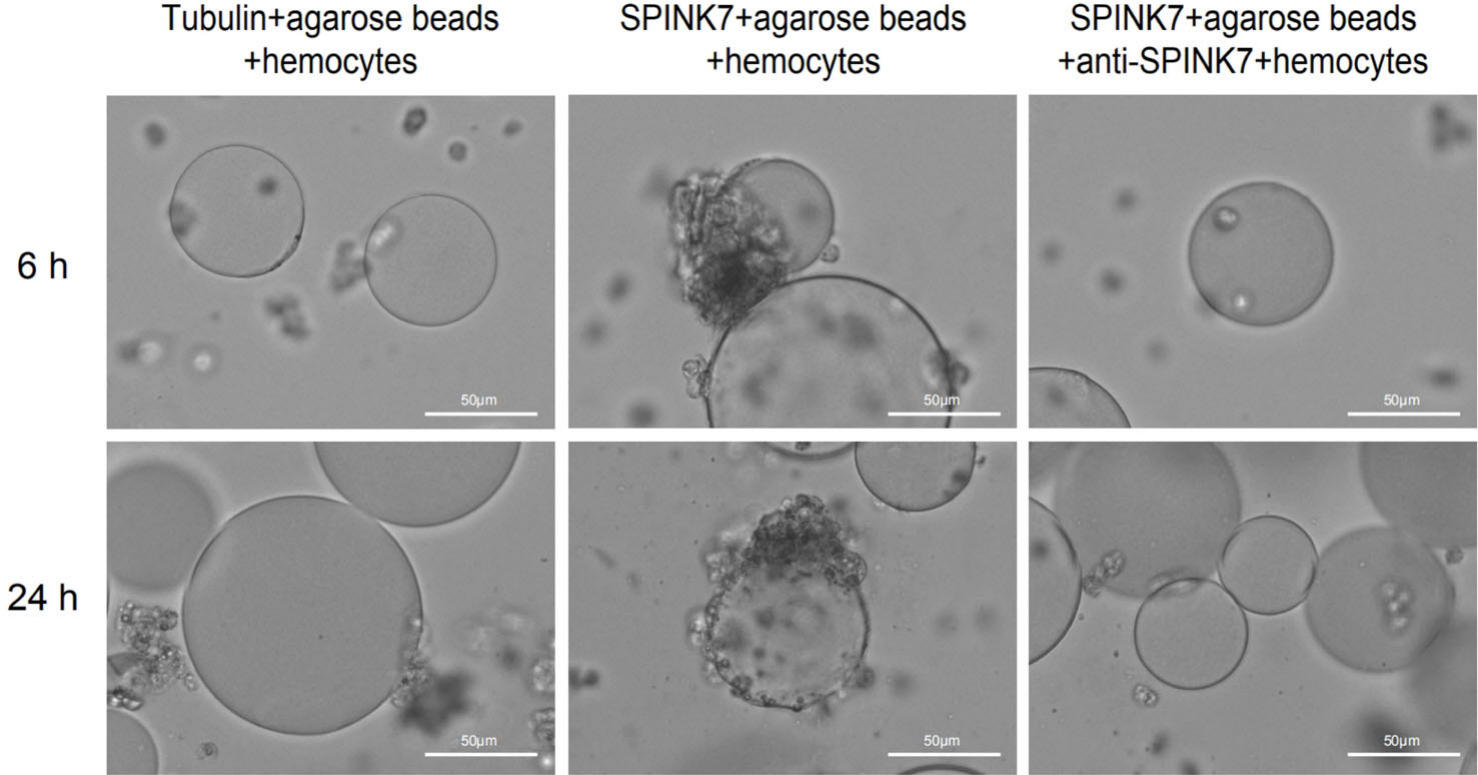
Encapsulation and melanization of SPINK7-coated beads by hemocytes. Nickel agarose beads (80-100 beads) coated with SPINK7 or tubulin (as a control protein) were incubated with *B. mori* larval hemocytes. Encapsulation and melanization of the protein-coated beads were observed by microscopy after 6 and 24 h incubation. SPINK7-coated beads were encapsulated and melanized by *B. mori* hemocytes. Antibody specific for SPINK7 blocks hemocyte encapsulation of the SPINK7-coated beads.

Generally, hemocytes fight against small invaders such as bacteria by phagocytosis, whereas they attack large invaders by nodulation and encapsulation. The process of nodulation and encapsulation is similar, except that the object volume of encapsulation is larger than that of nodulation (34–36). To investigate whether SPINK7 was involved in cellular immunity, we performed *in vitro* assays using protein-coated agarose beads. Obvious encapsulation and melanization of beads by *B. mori* hemocytes was observed when beads was coated with SPINK7 protein (**Figure 8**). As control, beads coated with tubulin protein were not encapsulated and melanized by *B. mori* hemocytes (**Figure 8**). When SPINK7 beads were pre-incubated with rabbit anti-SPINK7 antibody and then incubated with *B. mori* hemocytes, the encapsulation and melanization of hemocyte was effectively blocked (**Figure 8**). These results suggested that SPINK7 can promote hemocyte encapsulation and melanization through direct interactions with hemocyte surface molecules.

### SPINK7 Induces Hemocytes to Aggregate and Entrap Fungi

**Figure 9:**
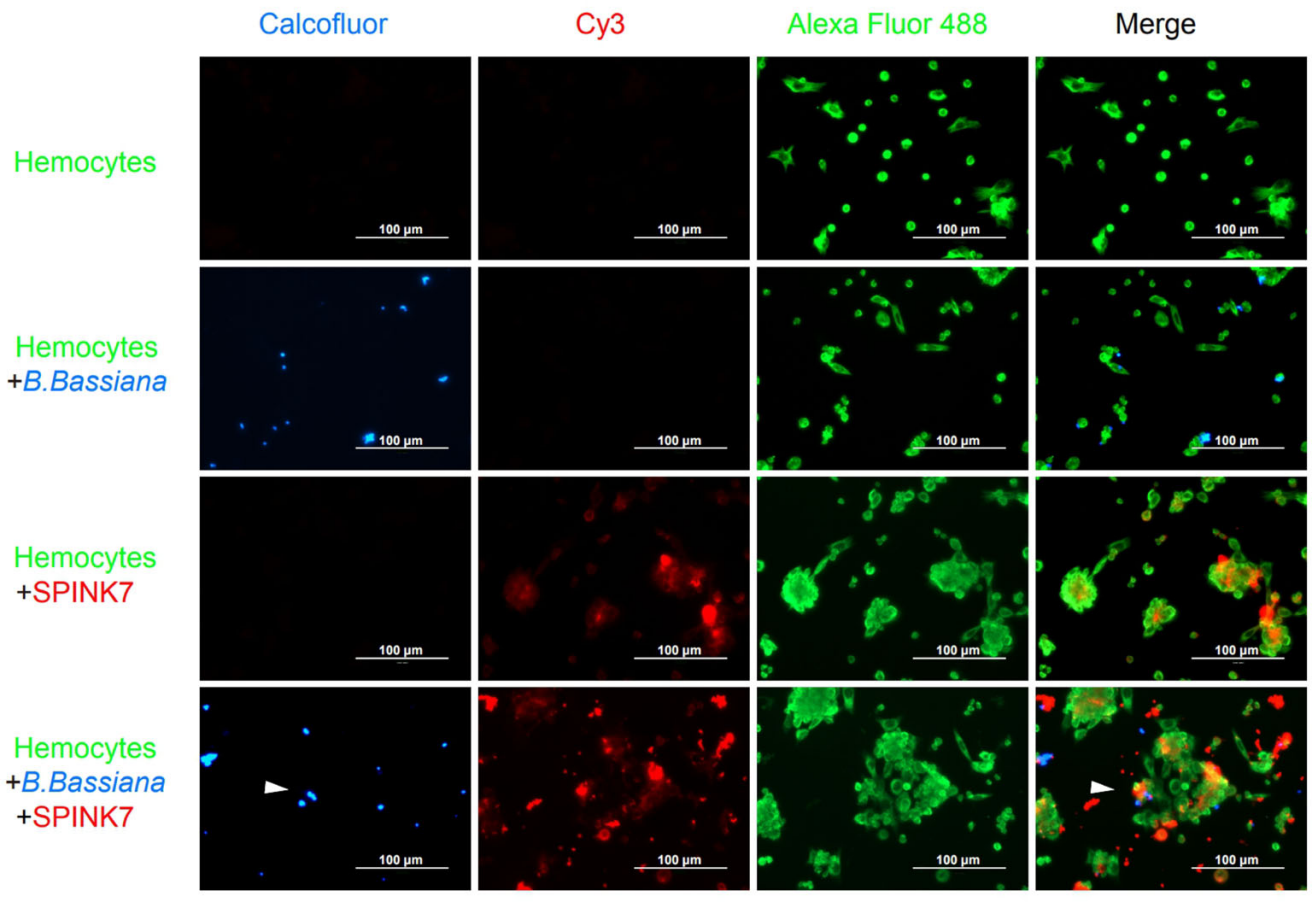
Immunofluorescence visualization of SPINK7 in *B. mori* hemocyte immunity. Overlay experiments were performed on hemocyte monolayers prepared by seeding 5×10^3^ hemocytes collected in phenylthiourea onto poly-L-lysine-treated glass slides. About 10 μL SPINK7 (5 μg μL^-1^) and 10 μL heat-killed *B. bassiana* spores (1×10^5^ conidia mL^-1^), which were labeled by Calcofluor White M2R (blue), were added to hemocyte monolayers and incubated at 25°C for 2 h. Slides were incubated with anti-SPINK7 and anti-tubulin antibodies, followed by Cy3-labeled goat anti-rabbit (red) and Alexa Fluor 488-labeled goat anti-mouse (green) antibodies.

**Figure 10:**
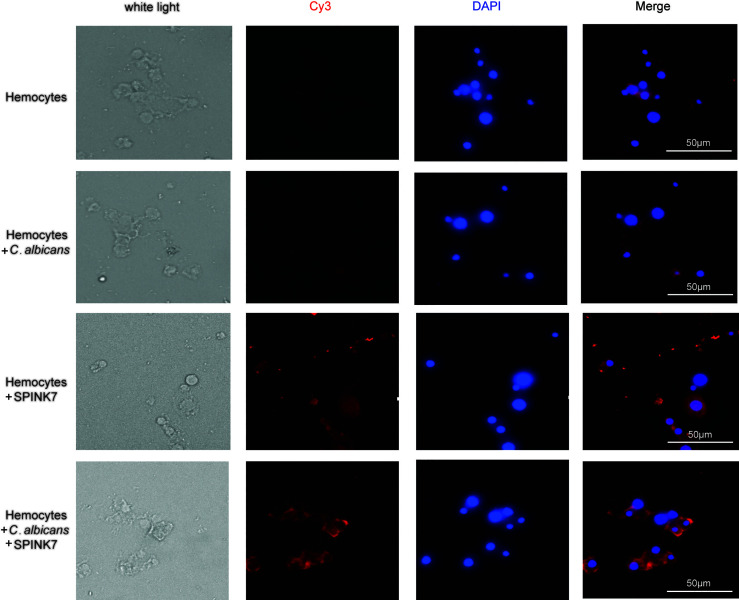
Immunofluorescence visualization of SPINK7 in *B. mori* hemocyte immunity. Overlay experiments were performed on hemocyte monolayers prepared by seeding 5×10^3^ hemocytes collected in phenylthiourea onto poly-L-lysine-treated glass slides. About 10 μL SPINK7 (5 μg μL^-1^) and 10 μL heat-killed *C. albicans* (1×10^5^ conidia mL^-1^) were added to hemocyte monolayers and incubated at 25°C for 2 h. Slides were incubated with anti-SPINK7, followed by Cy3-labeled goat anti-rabbit (red) antibodies.

Immunofluorescence was furtherly used to detect the interaction between SPINK7 and hemocytes (**Figure 9**). Hemocytes were scattered under normal conditions. Adding of heat-killed *B. bassiana* spores, labeled by Calcofluor White M2R (31), could not induce hemocyte aggregation, whereas SPINK7 addition promoted hemocyte aggregation and nodulation (**Figure 9**). After adding both SPINK7 and *B. bassiana* spores, SPINK7 promoted hemocytes to aggregate and bind to *B. bassiana* spores (**Figure 9**). We also performed immunofluorescence experiment with *C. albicans*, and found that SPINK7 could also promoted hemocytes to aggregate and bind to *C. albicans* (**Figure 10**).

To verify whether SPINK7 really enhance cellular immunity, we used BSA and heat-denatured SPINK7 (hdSPINK7) as the control. The results showed that BSA and hdSPINK7 did not bind to hemocytes and could not promote hemocyte aggregation (**Figure S2**). These results indicated that SPINK7 could bind to both fungi and hemocytes, and promotes nodulation.

## Discussion

Among numerous proteins in the hemolymph, nutrient-storage proteins and protease inhibitors are very abundant (4). At least seven protease inhibitor families with different domains were identified in the hemolymph, including serpin, TIL, Kazal, ITI, kunitz, WAP, and amfpi (4, 6). Previous studies revealed that protease inhibitors with TIL and kunitz domains play antifungal roles in silkworms (23, 24, 27). Many studies have also found that serine protease inhibitors with Kazal domain (SPINKs) were also involved in animal immunity against bacterial and fungi (18, 19, 37). This study focused on the silkworm SPINK7, and revealed its function in the cellular immunity.

We investigated whether SPINK7 expression changed after fungal induction, and found that SPINK7 was upregulated at various time points. By investigating the antifungal activity of SPINK7, we found it inhibited spore growth of three fungi species, including *C. albicans*, *S. cerevisiae*, and *B. bassiana*. Previous studies revealed that some protease inhibitors could inhibit fungal growth by inhibiting the activity of fungal proteases (22, 38–40). Thus, we examined the inhibitory activity of SPINK7 on fungi-derived proteases, and found that SPINK7 had weak inhibitory activity against proteinase K, a protease secreted by *Tritirachium album limber*. We speculated that SPINK7 might have a more efficient way to defend against fungi than relying solely on fungal protease inhibition.

For humoral immunity, PAMPs recognition in silkworms could rapidly amplify the immune signal by a cascade reaction of CLIP serine proteases (41, 42). Since SPINK7 showed a weak inhibitory effect on serine proteases, SPINK7 was unlikely to participate in humoral immunity as a cascade inhibitor. *C. albicans* has been reported to infect humans and silkworms (43), and *B. bassiana* is an important entomopathogenic fungus (42, 44, 45). Thus, we tested SPINK7 binding to these two fungi, and found that SPINK7 could bind with them, slightly break their cell membrane and have weak inhibition against their growth. Further, we found that SPINK7 was able to bind to β-D-glucan on the fungal cell wall, but SPINK7 does not bind mannan. Generally, N-linked glycosylation in insects contains abundant mannose (45, 46). Thus, SPINK7 recognized β-D-glucan rather than manman could avoid causing aggregation of own glycoproteins.

The function of SPINK7 is very similar to that of some humoral pattern recognition receptors that bind LPS, PGN and β-D-glucan associated with bacteria and fungi (34). These humoral pattern recognition receptors include hemolin, LPS-binding protein, Gram-negative bacteria recognition protein (GNBPs), soluble PGRPs (PGRP-SA and PGRP-SD), soluble forms of Down’s syndrome cell adhesion molecule (Dscam), and complement-like Tep proteins (34, 47–50). Because SPINK7 was expressed exclusively in hemocytes, we speculated that SPINK7 may be involved in cellular immunity. SPINK7 was able to recognize invaders and initiated hemocyte adhesion to the invaders. In this study, when SPINK7 was incubated with *B. bassiana*, we observed SPINK7-mediated nodulation of hemocytes, and when it was incubated with agarose beads, we observed SPINK7-mediated encapsulation of hemocytes. Encapsulation is usually followed by melanization, which may contribute to killing of the encapsulated invaders (51–54).

Overall, we found that SPINK7 was synthesized in hemocytes and secreted into the serum. SPINK7 was strongly upregulated in hemocytes after fungal challenge. Additionally, SPINK7 was able to bind to fungi through β-D-glucan on the cell wall and inhibited fungal growth. Finally, SPINK7 combined both fungi and hemocytes, caused hemocytes to aggregate, adhered to fungi and engulfed them. These reaction process were accompanied by melanization.

## Data Availability Statement

The datasets presented in this study can be found in online repositories. The names of the repository/repositories and accession number(s) can be found in the article/**Supplementary Material**.

## Author Contributions

ZD, QX, and PZ contributed to conception and design of the study. ZD and LA wrote the manuscript. ZD, LA, ML, MT, HC, XH, YH, GS, XZ, and YZ performed the research and analyzed the data. All authors contributed to the article and approved the submitted version.

## Funding

This work was supported by the Natural Science Foundation of Chongqing (cstc2020jcyj-cxttX0001), and Chongqing Research Program of Basic Research and Frontier Technology (cstc2019jcyj-msxmX0272).

## Conflict of Interest

The authors declare that the research was conducted in the absence of any commercial or financial relationships that could be construed as a potential conflict of interest.

## Publisher’s Note

All claims expressed in this article are solely those of the authors and do not necessarily represent those of their affiliated organizations, or those of the publisher, the editors and the reviewers. Any product that may be evaluated in this article, or claim that may be made by its manufacturer, is not guaranteed or endorsed by the publisher.

## Supplementary Material

Supplementary Figure 1 Analysis of purified SPINK7 by SDS-PAGE and western blot. Proteins were separated on 12% polyacrylamide gel. The BeyoColor pre-stained color protein ladder (6.5–240 kDa, Beyotime, China) and ExcelBand 3-color regular range protein Marker (10–180 kDa, Solarbio, China) were used as molecular markers.

Supplementary Figure 2 Immunofluorescence visualization of SPINK7 in *B. mori* hemocyte immunity. Overlay experiments were performed on hemocyte monolayers prepared by seeding 5×10^3^ hemocytes collected in phenylthiourea onto poly-L-lysine-treated glass slides. About 10 μL SPINK7 (5 μg μL^-1^) and 10 μL heat-killed *B. bassiana* spores (1×10^5^ conidia mL^-1^), which were labeled by Calcofluor White M2R (blue), were added to hemocyte monolayers and incubated at 25°C for 2 h. We used BSA (5 μg μL^-1^) and heat-denatured SPINK7 (hdSPINK7) (5 μg μL^-1^) to replace SPINK7 as a control experiment. Slides were incubated with anti-SPINK7 and tubulin antibodies followed by Cy3-labeled goat anti-rabbit (red) and Alexa Fluor 488-labeled goat anti-mouse (green). Hemocytes were observed at 400× magnification.
