# Supplementary figures and images for: SPINK7 Recognizes Fungi and Initiates Hemocyte-Mediated Immune Defense Against Fungal Infections
Source: Front Immunol. 2021 Sep 17;12:735497. doi: 10.3389/fimmu.2021.735497 (PMC8484702; doi:10.3389/fimmu.2021.735497)

Figure S1

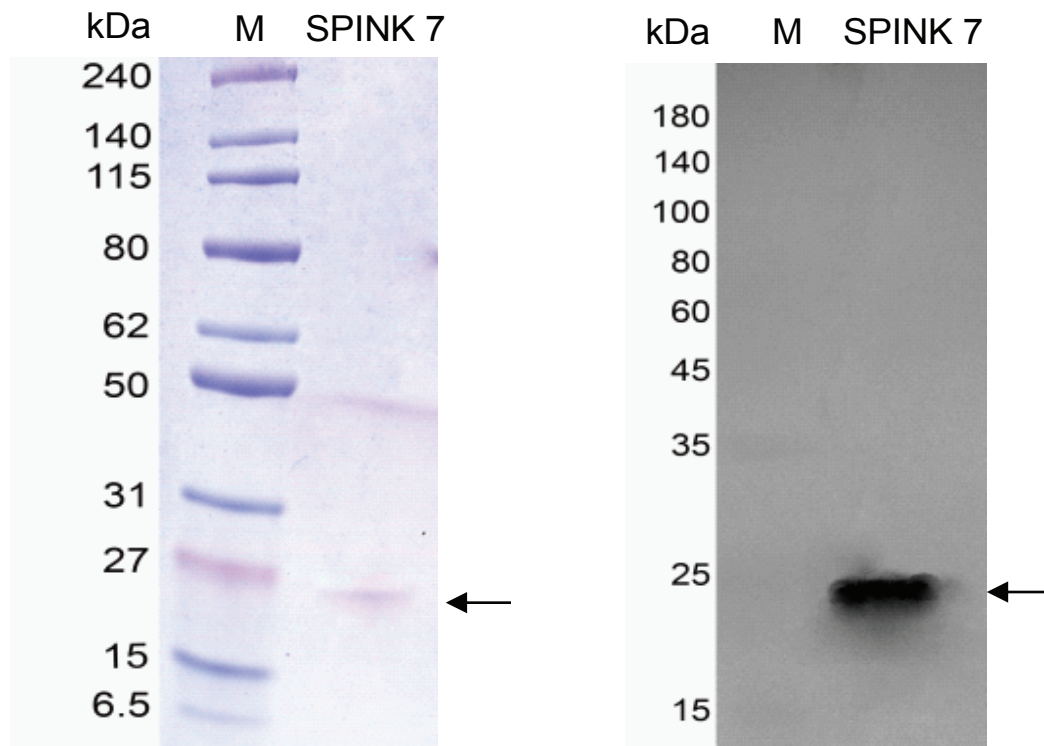

Supplement: Supplementary Figure 1 — Analysis of purified SPINK7 by SDS-PAGE and western blot. Proteins were separated on 12% polyacrylamide gel. The BeyoColor pre-stained color protein ladder (6.5–240 kDa, Beyotime, China) and ExcelBand 3-color regular range protein Marker (10–180 kDa, Solarbio, China) were used as molecular markers. [file Image_1.pdf]

Figure S2

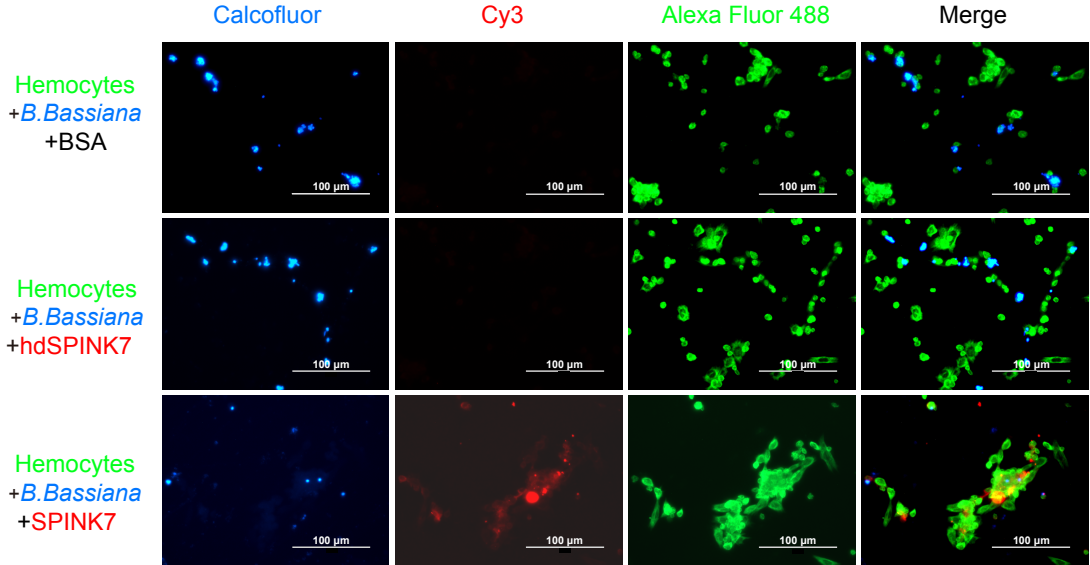

Supplement: Supplementary Figure 2 — Immunofluorescence visualization of SPINK7 in B. mori hemocyte immunity. Overlay experiments were performed on hemocyte monolayers prepared by seeding 5×10^3^ hemocytes collected in phenylthiourea onto poly-L-lysine-treated glass slides. About 10 μL SPINK7 (5 μg μL-1) and 10 μL heat-killed B. bassiana spores (1×10^5^ conidia mL-1), which were labeled by Calcofluor White M2R (blue), were added to hemocyte monolayers and incubated at 25°C for 2 h. We used BSA (5 μg μL-1) and heat-denatured SPINK7 (hdSPINK7) (5 μg μL-1) to replace SPINK7 as a control experiment. Slides were incubated with anti-SPINK7 and tubulin antibodies followed by Cy3-labeled goat anti-rabbit (red) and Alexa Fluor 488-labeled goat anti-mouse (green). Hemocytes were observed at 400× magnification. [file Image_2.pdf]
